# AFA-Recur: an ESC EORP AFA-LT registry machine-learning web calculator predicting atrial fibrillation recurrence after ablation

**DOI:** 10.1093/europace/euac145

**Published:** 2022-08-25

**Authors:** Andrea Saglietto, Fiorenzo Gaita, Carina Blomstrom-Lundqvist, Elena Arbelo, Nikolaos Dagres, Josep Brugada, Aldo Pietro Maggioni, Luigi Tavazzi, Josef Kautzner, Gaetano Maria De Ferrari, Matteo Anselmino

**Affiliations:** Division of Cardiology, Department of Medical Sciences, ‘Città della Salute e della Scienza di Torino’ Hospital, University of Turin, Turin, Italy; Cardiology Unit, J Medical, Turin, Italy; Department of Medical Science and Cardiology, Uppsala University, Uppsala, Sweden; Department of Cardiology, Cardiovascular Institut, Hospital Clinic de Barcelona, Universitat de Barcelona, Barcelona, Spain; Institut d’Investigació August Pi iSunyer (IDIBAPS), Barcelona, Spain; Centro de Investigación Biomédica en Red de Enfermedades Cardiovasculares (CIBERCV), Madrid, Spain; Department of Electrophysiology, Heart Center Leipzig at University of Leipzig, Leipzig, Germany; Hospital Clínic Pediatric Arrhythmia Unit, Cardiovascular Institute, Hospital Sant Joan de Déu University of Barcelona, Barcelona, Spain; EURObservational Research Programme (EORP), European Society of Cardiology, Sophia-Antipolis, France; ANMCO Research Centre, Florence, Italy; Cardiovascular Department, Maria Cecilia Hospital, GVM Care & Research, Cotignola, Italy; Department of Cardiology, Institute for Clinical and Experimental Medicine (ΙΚΕΜ), Prague, Czech Republic; Division of Cardiology, Department of Medical Sciences, ‘Città della Salute e della Scienza di Torino’ Hospital, University of Turin, Turin, Italy; Division of Cardiology, Department of Medical Sciences, ‘Città della Salute e della Scienza di Torino’ Hospital, University of Turin, Turin, Italy

**Keywords:** Atrial fibrillation, Transcatheter ablation, Recurrence, Predictors, Machine learning

## Abstract

**Aims:**

Atrial fibrillation (AF) recurrence during the first year after catheter ablation remains common. Patient-specific prediction of arrhythmic recurrence would improve patient selection, and, potentially, avoid futile interventions. Available prediction algorithms, however, achieve unsatisfactory performance. Aim of the present study was to derive from ESC-EHRA Atrial Fibrillation Ablation Long-Term Registry (AFA-LT) a machine-learning scoring system based on pre-procedural, easily accessible clinical variables to predict the probability of 1-year arrhythmic recurrence after catheter ablation.

**Methods and results:**

Patients were randomly split into a training (80%) and a testing cohort (20%). Four different supervised machine-learning models (decision tree, random forest, AdaBoost, and *k*-nearest neighbour) were developed on the training cohort and hyperparameters were tuned using 10-fold cross validation. The model with the best discriminative performance on the testing cohort (area under the curve—AUC) was selected and underwent further optimization, including re-calibration. A total of 3128 patients were included. The random forest model showed the best performance on the testing cohort; a 19-variable version achieved good discriminative performance [AUC 0.721, 95% confidence interval (CI) 0.680–0.764], outperforming existing scores (e.g. APPLE score: AUC 0.557, 95% CI 0.506–0.607). Platt scaling was used to calibrate the model. The final calibrated model was implemented in a web calculator, freely available at http://afarec.hpc4ai.unito.it/.

**Conclusion:**

AFA-Recur, a machine-learning-based probability score predicting 1-year risk of recurrent atrial arrhythmia after AF ablation, achieved good predictive performance, significantly better than currently available tools. The calculator, freely available online, allows patient-specific predictions, favouring tailored therapeutic approaches for the individual patient.

## Graphical Abstract

**Graphical Abstract:**
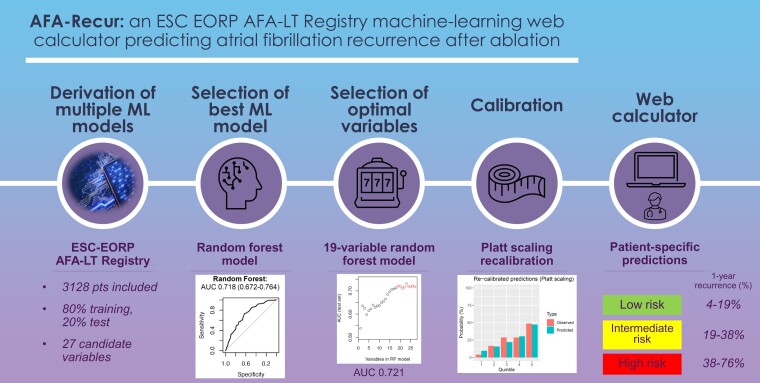

**What’s new?**

- Supervised machine-learning (ML) algorithms based on pre-procedural, easily accessible, clinical variables from the ESC-EHRA Atrial Fibrillation Ablation Long-Term Registry were used to derive a scoring system to predict the probability of 1-year arrhythmic recurrence after atrial fibrillation catheter ablation.
- The best model (random forest) showed good discriminative performance on the testing cohort (area under the curve of 19-variable version: AUC 0.721, 95% confidence interval 0.680–0.764).
- The final calibrated model was implemented in a web calculator, freely available online, allowing patient-specific predictions and tailored therapeutic decisions.

## Introduction

Atrial fibrillation (AF) is the most common arrhythmia encountered in daily clinical practice, with a currently estimated prevalence in the adult population ranging from 2 to 4%. Atrial fibrillation–associated mortality and morbidity burden is expected to rise in the forthcoming years, as recent epidemiological projections foresee a doubling in prevalence by 2050.^1^

A rhythm-control approach is recommended for symptoms and quality of life^1^ improvement, and, as recently documented in the EAST-AFNET 4 trial,^2^ it may also achieve a reduction in adverse cardiovascular outcomes, at least in case the arrhythmia is present since <12 months. In this respect, catheter ablation is a well-established option, achieving, compared with antiarrhythmic drugs, superior efficacy in maintaining sinus rhythm,^3^ also as first-line option.^4^

However, recurrent AF after catheter ablation remains relatively common and multiple procedures are often required.^5,6^ Freedom from recurrent AF improves the benefit deriving from AF catheter ablation in terms of symptom relief and possibly also of serious adverse events, given the known association of sinus rhythm maintenance with survival.^7^ The need of better patient selection is, therefore, mandatory. Different scoring systems have been proposed to predict recurrent AF after catheter ablation,^8^ however, discriminatory abilities of the models are largely suboptimal and most studies did not perform calibration.

The machine-learning (ML) field is steadily growing and several examples exist of ML-derived scores outperforming traditional risk scores in predicting cardiovascular outcomes.^9,10^ Aim of the present study is to derive from the prospective, multicentre, multinational European Society of Cardiology (ESC)-EHRA Atrial Fibrillation Ablation Long-Term Registry (AFA-LT), an ML algorithm based on pre-procedural, easily accessible clinical variables, to predict the risk of 1-year recurrence of AF after catheter ablation, as part of a structured management of AF in accordance with recently published AF guidelines.^1^

## Methods

### Atrial Fibrillation Ablation Long-Term Registry

The ESC-EHRA AFA-LT is a prospective, multicentre, observational registry of consecutive patients undergoing an AF ablation procedure at 104 centres in 27 countries within the ESC. Participating centres enrolled consecutive patients scheduled for AF ablation between April 2012 and April 2015, following them up for 1 year. Atrial fibrillation was defined as paroxysmal or persistent according to 2010 ESC Guidelines definition.^11^ All patients gave written informed consent before study enrolment. Further details regarding the registry may be found in the original publication.^12^

### Outcome assessment

The investigated outcome was 1-year recurrence, defined as an electrocardiographically documented episode of AF or atrial flutter/tachycardia lasting at least 30 s after a 3-month blanking period from the ablation procedure. As detailed in the original AFA-LT registry publication, 1-year (median 12.4 months, interquartile range 11.9–13.4 months) follow-up evaluation was performed by an in-person clinical visit in 52.8%, a telephone contact in 44.2% and a contact with the patient’s general practitioner in 3.0% of the cases, respectively. During the registry period, strategies for arrhythmia recurrence detection included periodical clinical visits with electrocardiogram (EKG; 78.4%) and 24-h Holter monitoring (64.5%), according to caring physician’s discretion. Trans-telephonic monitoring and implanted monitoring systems were used in 3.4% of the cases, respectively. Overall, at least one EKG was performed in 86% of the patients and 82% had at least one physical evaluation during the 12-month follow up.

### Study inclusion criteria

Patients from the ESC-EHRA AFA-LT registry were included in the present study provided that: (i) ablation was performed; (ii) AF type was explicitly specified (paroxysmal or persistent); (iii) 1-year follow-up data regarding arrhythmic recurrences were available.

### Potential predictors and data pre processing

The following pre-procedural, easily available, covariates (based on personal history, clinical data, and echocardiographic assessment) were considered as potential candidate variables for the ML models training: age, gender, body mass index (BMI), estimated glomerular filtration rate (CKD-EPI formula were used), smoker status (active, former, never), hypertension, diabetes, dyslipidaemia, history of heart failure, coronary artery disease, structural heart disease (valvular heart disease, dilated cardiomyopathy, hypertrophic cardiomyopathy), previous stroke/transient ischemic attack, presence of cardiac rhythm device (either pacemaker, implantable cardioverter defibrillator, or cardiac resynchronization therapy), hyperthyroidism, peripheral artery disease, chronic obstructive pulmonary disease, obstructive sleep apnoea, CHA2DS2-VASc score, AF type (paroxysmal or persistent), history of atrial flutter, previous failed antiarrhythmic therapy, pre-procedural sinus rhythm, abnormal EKG (one or more of the following: atrioventricular block, bundle branch block, Q waves, ST-T abnormalities, and corrected QT > 460 ms), type of procedure (first ablation or re-do procedure), left ventricular ejection fraction (LVEF; %), left atrial (LA) anteroposterior diameter (mm), left ventricular end-diastolic volume (LVEDV; mL). Categorical variables are presented as numbers and percentages, while continuous variables as mean and standard deviation. Missing predictors were imputed using a *k*-nearest neighbour imputation (kNN) technique, with *k* = 5.

### Score derivation and validation

The original dataset was randomly split into a training (80%) and testing (20%) cohort. As a preliminary step, a standard backward stepwise logistic regression model was fitted on the training cohort and run on the testing cohort with poor outcome (see Supplementary material online, *Figure S1*). Therefore, four different supervised ML classifiers were fitted on the training cohort: decision tree (DT), random forest (RF), AdaBoost (ADA), and kNN. Model hyperparameters were optimized using 10-fold cross validation, fitting the final model with the set of tuning parameters which maximized the mean area under the curve (AUC) across the cross-validation samples. Discrimination of the four tuned models, in terms of AUC, was evaluated in the testing cohort. The model with the best AUC in the testing cohort was chosen as the model of interest, while other models were discarded. Variable importance was then computed for the chosen ML classifier using a filter-based approach. The change in AUC in the testing cohort was then evaluated progressively reducing the number of predictors in the chosen ML model, according to the previously computed variable importance ranking. In order to ensure proper discrimination, while at the same time, limiting model complexity in terms of number of predictors, the model with the best trade-off between discrimination (AUC) and complexity (number of predictors) was selected. The discriminatory ability of the final model in the testing cohort was then compared against the most known score, the APPLE score.^13^ Finally, model calibration was assessed on the testing cohort using reliability diagram and Hosmer–Lemeshow test. Platt scaling was performed to re-calibrate model predictions. Frequency distribution of the predicted re-calibrated probabilities and quintile analysis were also computed. The first two quintiles were considered ‘low’, the third and the fourth quintile ‘intermediate’, while the last quintile ‘high’ risk groups in terms of recurrence probability. The final calibrated ML model was used to implement a web-risk calculator.

All analyses were performed independently at our centre using R software version 4.0.0 (R Foundation for Statistical Computing, Vienna, Austria). In particular, *caret* package (https://cran.r-project.org/web/packages/caret/caret.pdf) was used to perform model training and hyperparameter optimization, while *shiny* package (https://shiny.rstudio.com/) was used to build the web calculator. A *P*-value <0.05 was considered statistically significant.

## Results

Overall, 3128 patients from the ESC-EHRA AFA-LT registry satisfied the inclusion criteria and were analysed. *Table 1* reports main clinical characteristics. Mean age was 58 ± 10 years, and 68.7% were males. Mean CHA_2_DS_2_-VASc score was 1.58 ± 1.32 and 20.6% had history of heart failure. Atrial fibrillation was persistent in 31.9% of the patients. The majority of the ablation procedures were performed with radiofrequency as energy source (83%).

**Table 1:** Baseline clinical variables, stratified by AF recurrence during 1-year follow up

Variables	Total (*n* = 3128)	Arrhythmic recurrence		
		No (*n* = 2331)	Yes (*n* = 797)	*P*-value
Age (years)	58.09 (10.28)	57.99 (10.27)	58.38 (10.30)	0.361
Male gender (%)	2148 (68.7)	1604 (68.8)	544 (68.3)	0.804
BMI (kg/m^2^)	28.40 (4.46)	28.29 (4.45)	28.74 (4.45)	0.017
eGFR CKDEPI (mL/min/1.73 m^2^)	80.80 (18.47)	81.62 (18.30)	78.47 (18.77)	<0.001
Heart failure (%)	431 (20.6)	298 (19.5)	133 (23.6)	0.046
CAD (%)	367 (17.9)	264 (17.6)	103 (18.4)	0.730
Structural heart disease (%)	535 (25.5)	379 (24.8)	156 (27.5)	0.230
Stroke/TIA (%)	96 (3.1)	68 (2.9)	28 (3.5)	0.476
Device carrier (%)	138 (4.4)	87 (3.7)	51 (6.4)	0.002
Smoker status (%)				0.066
ȃFormer (≥1 month)	588 (19.7)	417 (18.8)	171 (22.4)	
ȃNo	2082 (69.9)	1573 (71.0)	509 (66.7)	
ȃYes	309 (10.4)	226 (10.2)	83 (10.9)	
Diabetes (%)	301 (9.7)	220 (9.5)	81 (10.2)	0.599
Hypertension (%)	1680 (53.9)	1221 (52.7)	459 (57.6)	0.019
Dyslipidaemia (%)	988 (32.2)	739 (32.4)	249 (31.8)	0.809
Hyperthyroidism (%)	72 (2.3)	45 (2.0)	27 (3.5)	0.026
PAD (%)	55 (1.8)	42 (1.8)	13 (1.6)	0.751
COPD (%)	69 (2.3)	49 (2.2)	20 (2.5)	0.616
Obstructive sleep apnoea (%)	105 (3.6)	80 (3.7)	25 (3.4)	0.762
CHA_2_DS_2_-VASc score	1.58 (1.32)	1.55 (1.32)	1.66 (1.33)	0.043
AF type (%)				0.005
ȃParoxysmal AF	2130 (68.1)	1620 (69.5)	510 (64.0)	
ȃPersistent AF	998 (31.9)	711 (30.5)	287 (36.0)	
AFL (%)	724 (24.1)	529 (23.6)	195 (25.7)	0.264
Previous failed antiarrhythmic drugs (%)	2782 (89.7)	2064 (89.3)	718 (90.8)	0.273
Baseline LVEF (%)	59.81 (8.54)	60.01 (8.49)	59.20 (8.65)	0.045
Baseline LVEDV (mL)	112.47 (30.62)	112.84 (30.17)	111.57 (31.74)	0.543
Baseline LA diameter (mm)	42.63 (6.67)	42.22 (6.64)	43.81 (6.61)	<0.001
Baseline sinus rhythm (%)	1968 (62.9)	1514 (65.0)	454 (57.0)	<0.001
Abnormal ECG (%)	1885 (60.3)	1362 (58.4)	523 (65.6)	<0.001
Re-do procedure (%)	674 (21.5)	490 (21.0)	184 (23.1)	0.240

AF, atrial fibrillation; AFL, atrial flutter; BMI, body mass index; CAD, coronary artery disease; eGFR, estimated glomerular filtration rate; LA, left atrium; LVEDV, left ventricular end-diastolic volume; LVEF: left ventricular ejection fraction; PAD, peripheral artery disease; TIA, transient ischaemic attack.

During 1-year follow up, 797 patients (25.8%) experienced at least one arrhythmic recurrence (23.9% within paroxysmal AF patients). Baseline clinical variables, stratified by the presence of a recurrence during follow up, are reported in *Table 1*. Patients with arrhythmic recurrence more likely had persistent AF, history of heart failure, impaired renal function, and presented higher CHA_2_DS_2_-VASc score and BMI when compared with patients without recurrences. Additionally, those with recurrence showed greater LA anteroposterior diameter and lower LVEF than those without.

**Figure 1:**
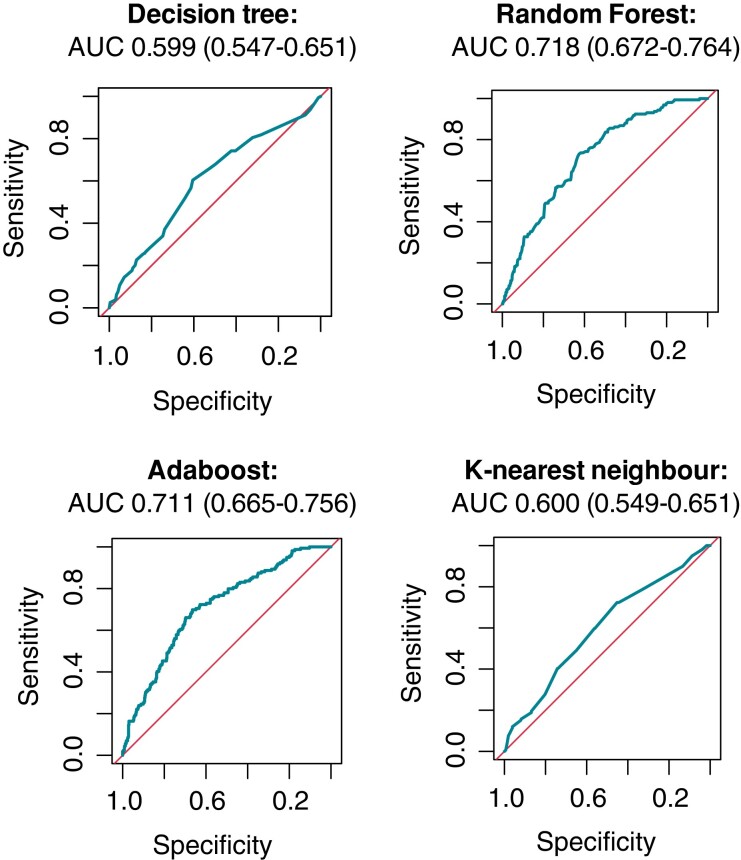
Receiver operator curve curves and corresponding AUC of the four evaluated machine-learning classifiers. AUC, area under the receiver operator curve.

**Figure 2:**
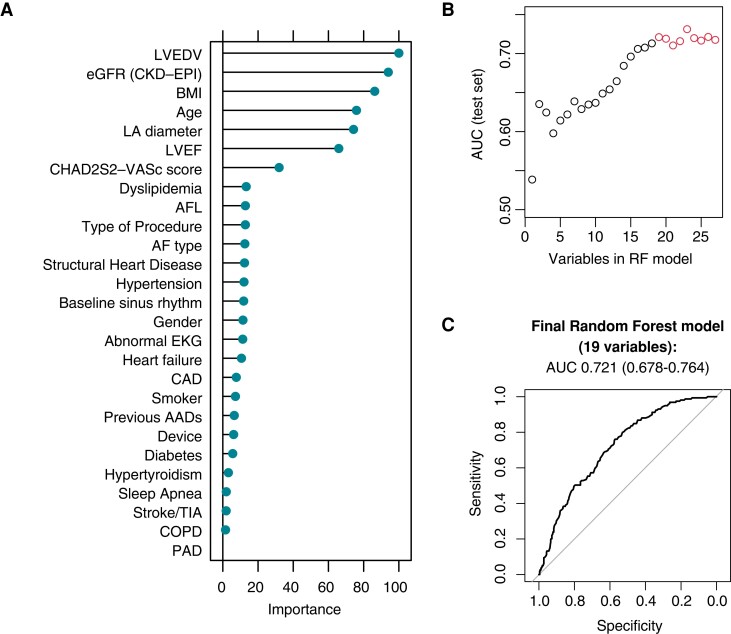
Random forest model selection. (*A*) Variable ranking for the 27 original predictors; (*B*) the number of included variables strengthens the model AUC reaching a plateau at 19; (*C*) ROC curve and corresponding AUC of the final 19-variable random forest model. AAD, antiarrhythmic drug; AFL, atrial flutter; AUC, area under the receiver operator curve; AFL, atrial flutter; BMI, body mass index; CAD, coronary artery disease; COPD, chronic obstructive pulmonary disease; LA, left atrium; LVEDV, left ventricular end-diastolic volume; LVEF: left ventricular ejection fraction; PAD, peripheral artery disease; RF: random forest; ROC: receiver operator curve.

Four different supervised ML classifiers (DT, RF, ADA, and kNN) were fitted and tuned on the training cohort (full details of the 10-fold cross validation on the training cohort for the optimally tuned models can be found in Supplementary material online, *Table S1*; AUC 0.722, interquartile range 0.691–0.739 for the RF model). Receiver operating characteristics with the corresponding AUC on the testing cohort for the different models are reported in *Figure 1*, with the RF model showing the best discriminative performance [AUC 0.718, 95% confidence interval (CI) 0.674–0.761] and thus chosen as the model of interest. *Figure 2A* reports variable ranking in the RF model. In order to ensure proper discrimination, while limiting model complexity, the change in AUC in the testing cohort was evaluated progressively reducing the number of predictors (starting by eliminating those with the lowest ranking). Simplified RF models were fitted (*K* features, with *K* ranging from 1 to 27), and the resulting AUC in the testing cohort was then plotted against the number of variables included in the model (*Figure 2B*). Given a plateau in the model discrimination was reached after 19 variables, a 19-variable RF model was chosen as the final ML classifier (*Table 2*).

**Table 2:** Variables included in the final random forest model

Included variables
LVEDV Estimated glomerular filtration rate (CKD-EPI formula) BMI Age LA anteroposterior diameter LVEF CHAD2DS2-VASc score Dyslipidaemia AFL Type of procedure (first procedure or re-do) Atrial fibrillation type (paroxysmal or persistent) Structural heart disease Hypertension Baseline sinus rhythm Gender Abnormal ECG Heart failure CAD Smoker

AFL, atrial flutter; BMI, body mass index; CAD, coronary artery disease; LA, left atrium; LVEDV, left ventricular end-diastolic volume; LVEF: left ventricular ejection fraction.

The AUC of the final model on the testing cohort was 0.721 (95% CI 0.680–0.764), outperforming the APPLE score in predicting outcomes in the testing cohort (AUC 0.557, 95% CI 0.506–0.607; see Supplementary material online, *Figure S2*).

**Figure 3:**
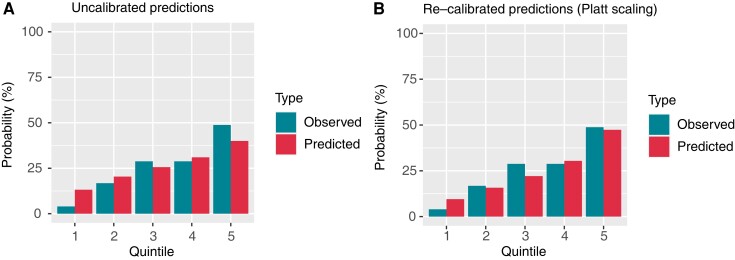
Random forest model calibration on the testing cohort. (*A*) Calibration plot before Platt scaling, showing uncalibrated predictions. (*B*) Calibration plot after Platt scaling, yielding improved calibration.

**Figure 4:**
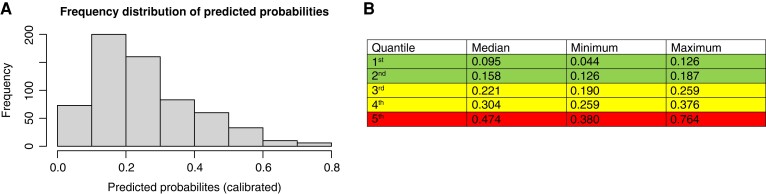
Predicted probability distribution and quantile analysis on the testing cohort. (*A*) Frequency distribution of the predicted probabilities of AF recurrence (after re-calibration with Platt scaling). (*B*) Quintile analysis of the predicted probability distribution; the first two quintiles are considered ‘low risk’ (green), the third and the fourth ‘intermediate-risk’ (yellow), while the last quintile represent ‘high risk’ (red) for AF recurrence after catheter ablation.

Eventually, given the uncalibrated predictions of the model (Hosmer–Lemeshow test *P* = 0.005), due to over-forecasting in the left lower quadrant of the reliability plot (*Figure 3A*), calibration was effectively performed by Platt scaling (*Figure 3B*; Hosmer–Lemeshow test *P* = 0.063). Frequency distribution of predicted probabilities after re-calibration is reported in *Figure 4A*. Quintile analysis (*Figure 4B*) defines three levels of progressively higher risk of recurrence: the first two quintiles comprise the ‘low’ (predicted probability range: 0.04–0.19), the third and the fourth quintiles the ‘intermediate’ (predicted probability range: 0.19–0.38), while the remaining upper quintile indicates the ‘higher risk group’ (predicted probability range: 0.38–0.76).

**Figure 5:**
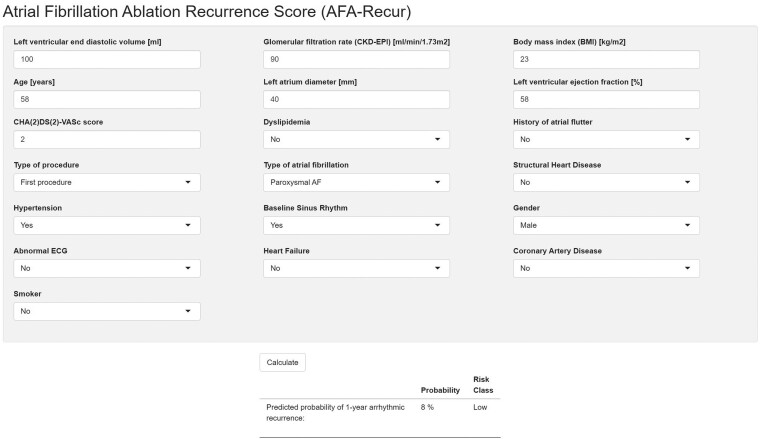
Screenshots of the freely available web calculator (http://afarec.hpc4ai.unito.it/), which allows the user to input predictor values to obtain a probability output of 1-year AF recurrence, as well as the associated risk class, for a specific patient.

The final re-calibrated RF model was ultimately implemented in a web calculator, freely available at http://afarec.hpc4ai.unito.it/, allowing the user to input predictor values to obtain the probability output of 1-year AF recurrence for a specific patient, as well as its associated risk class (*Figure 5*).

## Discussion

Catheter ablation has become a safe and well-established option in rhythm-control management of symptomatic AF patients.^1^ Although the recent Catheter Ablation vs. Antiarrhythmic Drug Therapy for Atrial Fibrillation (CABANA) trial did not reach formal statistical evidence in composite primary outcome, it revealed that catheter ablation reduces death or cardiovascular hospitalization (secondary endpoint^3^). In addition, clinical trials performed in patients with concomitant heart failure,^14^ and observational real-world evidences,^15^ suggest catheter ablation may be superior to medical therapy in reducing cardiovascular outcomes.

Efficacy of AF catheter ablation, however, remains an issue,^5,6^ with recurrence rates after a single procedure ranging between 20 and 45%.^16,17^ Part of the issue relates to inadequate durability of the ablation lesions, which may be solved by technological improvements. The remaining portion reflects, instead, the presence of complex myocardial substrate, the result of multiple clinical risk factors, and comorbidities. To avoid futile interventions, proper patient selection is therefore fundamental. Known risk factors associated with arrhythmia recurrence include persistent AF, enlarged left atrium, and presence of underlying structural cardiopathy.^18,19^ In this regard, several prognostic models combining different predictors have been proposed. However, a recent meta-analysis^8^ evaluating 13 prognostic models showed that the discriminatory ability of the models was suboptimal, with no model proving consistently good performance in predicting rhythm outcome. In addition, none of the thirteen models underwent internal validation, translating into an overly optimistic prediction of performance of the model when used in unknown populations. Furthermore, only two out of 13 models assessed calibration, lacking, in the majority of cases, an important step in model optimization.

In the present study, we derived and tested the first ML-based probability score of the risk of recurrent arrhythmic events in patients undergoing AF catheter ablation (AFA-Recur), based on the widest available, prospective, multicentre, multinational, observational registry of the ESC (ESC-EHRA AFA-LT). Machine learning is a rapidly evolving field, with increasing use in cardiovascular medicine.^20^ It broadly refers to analytical algorithms that iteratively learn from data, discovering hidden, but potentially relevant, associations without being explicitly programmed where to search. In fact, the main advantage of ML-based approach is that it takes into account multiple, complex, non-linear interactions between the various characteristics and comorbidities that constitute the full portrait of each patient, without the need of directly specifying variable interaction terms such as in traditional statistical approaches (e.g. logistic regression). Recent studies have shown that ML-based cardiovascular predictive modelling outperforms traditional risk scores,^9,21^ and have been assessed in several clinical scenarios.^22^ Our work strengthens this consideration; in fact, the present ML-based probability risk score shows good performance in predicting 1-year arrhythmic recurrence after catheter ablation, outperforming the most widely used existing risk score, the APPLE score.^13^

An additional novelty of the present score is that it is derived from a heterogeneous population, originating from 104 centres in 27 countries within the ESC. Differently from previous risk scores developed in highly selected populations, the multicentre, multinational nature of the ESC-EHRA AFA-LT registry grants the opportunity to capture the heterogeneity that exists between different centres and countries in terms of patient selection and procedural features, highly representative of a real-life scenario. Furthermore, the prospective nature of ESC-EHRA AFA-LT registry ensures that patients are not selected based on availability of predictors or outcome data.

### Limitations

Some limitations of the present study need to be addressed. First, since only a limited quote of patients were implanted with loop recorders after the procedure, brief, asymptomatic arrhythmia recurrences may have been missed in outcome adjudication. However, previous literature suggests that only a small percentage of patients clinically considered arrhythmia-free after catheter ablation may meet ablation failure definition (recurrent arrhythmia lasting >30 s) using long-term electrocardiographic monitoring.^23^ Additionally, being symptom control the main goal for AF ablation, brief asymptomatic arrhythmias may not represent a clear procedural failure.

Second, ESC-EHRA AFA-LT registry defined AF as paroxysmal or persistent according to 2010 ESC Guidelines definition,^11^ and definitions have changed in the subsequent guidelines, leading to a marked shift from persistent to paroxysmal AF. It should be noted, however, that a recent report hints that the original definition might provide a better separator to predict rhythm outcome after AF ablation.^24^

Finally, given the minority of patients undergoing cryoballoon ablation in this population (16%), the present score might mainly apply to patients undergoing radiofrequency ablation. In fact, although success rate by cryoballoon is equivalent to radiofrequency ablation,^25^ recurrence predictors between the two approaches might differ. The preponderance of patients undergoing radiofrequency ablation precluded subgroup analysis to potentially identify differential predictors of recurrence between different devices and energy sources.

## Conclusions

Based on the widest available, prospective, multicentre, multination observational registry of AF patients undergoing catheter ablation (ESC-EHRA AFA-LT registry), we derived and tested an ML-based probability score evaluating 1-year risk of recurrent arrhythmic events after ablation (AFA-Recur).

The freely available online calculator (http://afarec.hpc4ai.unito.it/) offers end users the possibility to predict, by inserting easily derived pre-procedural clinical variables, the patient-specific risk of recurrent atrial arrhythmias after ablation. The good discriminative performance achieved by the model enables tailored therapeutic approaches for the individual patient.

## Supplementary material

Supplementary material is available at *Europace* online.

## Supplementary Material

euac145_Supplementary_Data

## Data Availability

The data underlying this article were provided by ESC with permission.
