# Supplementary material for: AFA-Recur: an ESC EORP AFA-LT registry machine-learning web calculator predicting atrial fibrillation recurrence after ablation
Source: Europace. 2022 Aug 25;25(1):92–100. doi: 10.1093/europace/euac145 (PMC10103564; doi:10.1093/europace/euac145)
Supplement: euac145_Supplementary_Data [file euac145_supplementary_data.docx]

**AFA-Recur: an ESC EORP AFA-LT Registry machine-learning web calculator predicting atrial fibrillation recurrence after ablation**

*Supplementary Material*

**Committees and Investigators**

**EORP Oversight Committee**

Christopher Peter Gale, Chair, GB, Branko Beleslin, RS, Andrzej Budaj, PL, Ovidiu Chioncel, RO, Nikolaos Dagres, DE, Nicolas Danchin, FR, David Erlinge, SE, Jonathan Emberson, GB, Michael Glikson, IL, Alastair Gray, GB, Meral Kayikcioglu, TR, Aldo Maggioni, IT, Klaudia Vivien Nagy, HU, Aleksandr Nedoshivin, RU, Anna-Sonia Petronio, IT, Jolien Roos-Hesselink, NL, Lars Wallentin, SE, Uwe Zeymer, DE.

**AFA LT registry**

**Executive Committee**

Nikolaos Dagres, DE, Josep Brugada, ES, Elena Arbelo, ES, Luigi Tavazzi, IT, Carina Blomström Lundqvist, SE, Josef Kautzner, CZ, Aldo P. Maggioni, IT.

**Steering Committee (National Coordinators)**

Clemens Steinwender, AT, Alexandr Chasnoits, BY, Georges Mairesse, BE, Tosho Balabanski, BG, Josef Kautzner, CZ, Sam Riahi, DK, Mostafa Nawar, EG, Mervat Abul El Maaty, EG, Pekka Raatikainen, FI, Frederic Anselme, FR, Thorsten Lewalter, DE, Michalis Efremidis, GR, Laszlo Geller, HU, Ben Glover, CA, Roy Beinart, IL, Michael Glikson, IL, Fiorenzo Gaita, IT, Roin Rekvava, KZ, Oskars Kalejs, LV, Serge Trines, NL, Zbigniew Kalarus, PL, Mario Martins Oliveira, PT, Pedro Adragao, PT, Radu Ciudin, RO, Evgeny Mikhaylov, RU, Matjaz Sinkovec, SI, Julian Perez Villacastin, ES, Carina Blomström-Lundqvist, SE, Oleg Sychov, UA, Paul Roberts, GB.

**Investigators**

**AUSTRIA** *Graz* D Daniel Scherr; Martin Manninger; Bernadette Mastnak; *Innsbruck* Otamr Pachinger; Florian Hintringer; Markus Stühlinger; *Linz* Clemens Steinwender; **BELGIUM** *Yvoir* Olivier Xhaet; **BULGARIA** *Sofia* Tchavdar Shalganov; Milko Stoyanov; Mihail Protich; Vassil Traykov; Daniel Marchov; Genadi Kaninski; **BELARUS** *Minsk* Alexandr Chasnoits; **CZECH REPUBLIC** *Prague* Robert Cihak; *Hradec Kralove* Ludek Haman; **GERMANY** *Frankfurt* Boris Schmidt; K.R. Julian Chun; Laura Perrotta; Stefano Bordignon; *Hamburg* Roland Tilz; Stephan Willems; *Leipzig* Gerhard Hindricks; *Munich* Turgut Brodherr; Ilia S. Koutsouraki; Thorsten Lewalter; **DENMARK** *Aalborg* Sam Riahi; Bodil Ginnerup Sørensen; **EGYPT** *Cairo* Wagdi Galal; Amir AbdelWahab; S Sherif Mokhtar; **FINLAND** *Turku* Juha Lund; *Tampere* Pekka Raatikainen; **FRANCE** *Grenoble* Pascal Defaye; Peggy Jacon; Sandrine Venier; Florian Dugenet; *Saint Denis* Olivier Piot; Xavier Copie; Olivier Paziaud; Antoine Lepillier; *Saint Etienne* Antoine Da Costa; Cécile Romeyer-Bouchard; *Toulouse* Serge Boveda; Jean-Paul Albenque; Nicolas Combes; Stéphane Combes *Marseille* Ange Ferracci; André Pisapia; **GREECE** *Athens* Demosthenes Katritsis; Konstantinos Letsas; Kostas Vlachos; Louiza Lioni; *Thessaloniki* Vassilios P. Vassilikos; **HUNGARY** *Budapest* Laszlo Geller, Nándor Szegedi; Gábor Széplaki; Tamás Tahin; *Debrecen* Zoltan Csanadi; Gabor Sandorfi; Alexandra Kiss; Edina Nagy-Balo; *Szeged* Laszlo Saghy; **IRELAND** *Dublin* Benedict M. Glover; Joseph Galvin; Edward Keelan; **ISRAEL** *Ramat* Roy Beinart; Michael Glikson; Eyal Nof; **ITALY** *Acquaviva delle Fonti* Massimo Grimaldi; Federico Quadrini; Antonio Di Monaco; Federica Troisi; *Castellanza* Massimo Tritto; Elvira Renzullo; Antonio Sanzo; Domenico Zagari; *Cotignola* Carlo Pappone; *Crema* Pietro Maria Giovanni Agricola; *Milan* Paolo Della Bella; *Napoli* Giuseppe Stabile; Assunta Iuliano; *Pisa* Maria Grazia Bongiorni; *Roma* Leonardo Calo; Ermenegildo de Ruvo; Luigi Sciarra; *Turin* Matteo Anselmino; Fiorenzo Gaita; Federico Ferraris; *Varese* Roberto De Ponti; Raffaella Marazzi; Lorenzo A. Doni; **KAZAKHSTAN** *Almaty* Roin Rekvava; Anna Kim; **LATVIA** *Riga* Oskars Kalejs; **NETHERLANDS** *Breda* Sander Molhoek; *Groningen* Isabelle Van Gelder; Michiel Rienstra; *Leiden* Serge A. Trines; Marieke G. Compier; *Maastricht* Laurent Pison; Harry J. Crijns; Kevin Vernooy; Justin Luermans; *Rotterdam* Luc Jordaens; Natasja de Groot; Tamas Szili-Torok; Rohit Bhagwandien; *Zwolle* Arif Elvan; Thomas Buist; Pim Gal; **POLAND** *Lodz* Andrzej Lubinski; *Gdansk* Tomasz Krolak; *Katowice* Seweryn Nowak, Katarzyna Mizia-Stec; Anna Maria Wnuk-Wojnar; *Krakow* Jacek Lelakowski; *Szczecin* Jaroslaw Kazmierczak; *Poznan* Krzysztof Blaszyk; *Warszawa* Piotr Kulakowski; Jakub Baran; Grzegorz Opolski; Marek Kiliszek; Piotr Lodziński; Sonia Borodzicz; Paweł Balsam; Mariusz Pytkowski; Rafal Kuteszko; Jan Ciszewski; *Wroclaw* Artur Fuglewicz; *Zabrze* Zbigniew Kalarus; Aleksandra Woźniak; Karolina Adamczyk; **PORTUGAL** *Carnaxide* Pedro Adragao; *Lisbon* Pedro Cunha; **ROMANIA** *Iasi* Mihaela Grecu; Grigore Tinica; *Cluj-Napoca* Lucian Muresan; Radu Rosu; **RUSSIAN FEDERATION** *Kemerovo* Egor Khomenko; *Khanty- Mansiysk* Nikita Scharikov; *Krasnoyarsk* Dmitry Zamanov; Evgenii Kropotkin; *Novosibirsk* Evgeny Pokushalov; Alexander Romanov; Sevda Bayramova; *Saint-Petersburg* Evgeny N. Mikhaylov; Dmitry S. Lebedev; Anna V. Patsouk; Sergey Yashin; Dmitry Kryzhanovskiy; *Saransk* Vyacheslav Bazayev; *Surgut* Denis Morgunov; Ilya Silin; *Tomsk* Sergey Popov; *Tyumen* Vadim Kuznetsov; **SPAIN** *Alicante* Ignacio Gil Ortega; Juan Gabriel Martinez Martinez; *Badajoz* Manuel Doblado Calatrava; *Barcelona* Roger Villuendas Sabate; Lluis Mont Girbau; *Bilbao* Maria Fe Arcocha; Larraitz Gaztañaga; Estibaliz Zamarreño*; Granada* Miguel Álvarez; Rosa Macías; *Las Palmas de Gran Canaria* Federico Segura Villalobos; Juan Carlos Rodríguez Pérez; *Madrid* Nicasio Perez Castellano; Victoria Cañadas; Juan J Gonzalez Ferrer; David Filgueiras; Rafael Peinado; David Filqueiras-Rama; Alfonso Gómez Gallanti; Daniel Garófalo; *Pamplona* Naiara Calvo; Jose Manuel Rubio Campal; Pepa Sánchez-Borque; Juan Benezet-Mazuecos; Jorge Toquero Ramos; Fernandez Lozano; Victor Castro Urda; *Malaga* Alberto Barrera Cordero; Carmen Medina Palomo; Amalio Ruiz-Salas; Javier Alzueta; *Santander* Juan Jose Olalla Antolin; *Sevilla* Alonso Pedrote; Eduardo Arana-Rueda; Lorena García-Riesco; **SWEDEN** *Linköping* Anders Jönsson; *Lund* Pyotr Platonov; Fredrik Holmqvist; Ole Kongstad; Shiwen Yuan; *Umeå* Niklas Höglund; *Uppsala* Helena Malmborg; David Mörtsell; **SLOVENIA** *Ljubljana* Matjaz Sinkovec; Andrej Pernat; **UNITED KINGDOM** *Southampton* John Morgan; Paul Roberts; Elizabeth F. Greenwood; Lisa L. Fletcher; **UKRAINE** *Donetsk* Tetiana Kravchenko; *Kiev* Alexander Doronin; Maryna Meshkova; *Odessa* Iurii Karpenko; Alex Goryatchiy; Anna Abramova.

**Supplementary Tables**

**Supplementary Table 1. AUC summary statistics obtained by 10-fold cross validation on the training cohort of the optimally tuned models.**

| **Model** | Min. | 1st Quartile | Median | Mean | 3rd Quartile | Max. |
| --- | --- | --- | --- | --- | --- | --- |
| Decision Tree | 0.5396056 | 0.5831623 | 0.6073169 | 0.5977372 | 0.6154991 | 0.6425158 |
| Random Forest | 0.6411681 | 0.6907843 | 0.7217968 | 0.7101542 | 0.7385999 | 0.7544103 |
| Adaboost | 0.6300969 | 0.6883615 | 0.7162298 | 0.7052702 | 0.7319899 | 0.7583556 |
| KNN | 0.5162099 | 0.5568606 | 0.5881996 | 0.5860105 | 0.6271219 | 0.6422126 |

**Supplementary Figures**

**Supplementary Figure 1. ROC curve for standard stepwise backward logistic regression model applied on the testing cohort, yielding an** **AUC of** **0.578 (95% CI 0.527-0.629).** The resulting variables of the model after stepwise backward selection were: age, eGFR (CKD-EPI), heart failure, device, dyslipidemia, hyperthyroidism, LA diameter and abnormal EKG.

AUC: area under the receiver operator curve; EKG: electrocardiogram; LA: left atrium; ROC: receiver operator curve.

**
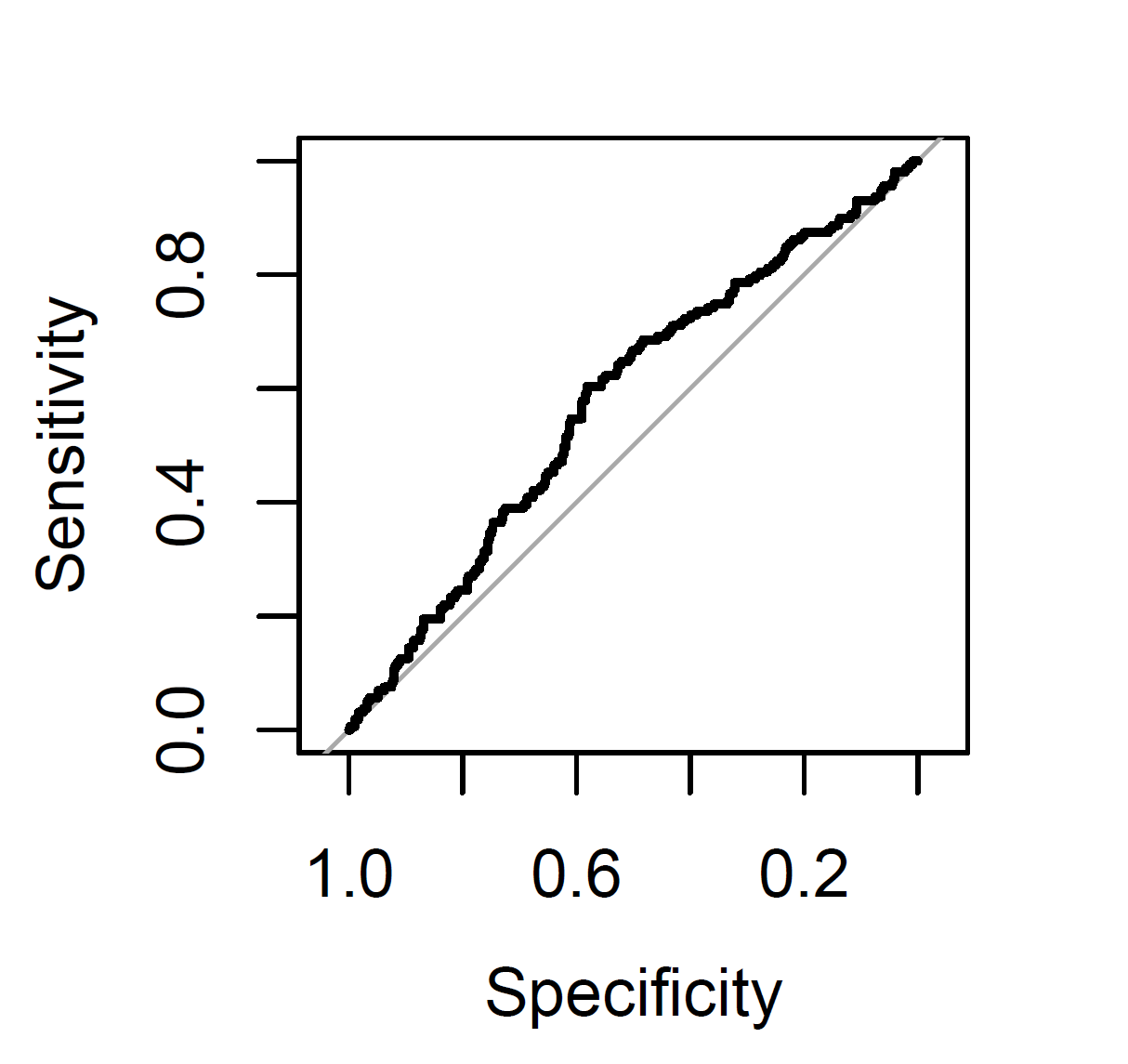
**

**Supplementary Figure 2. ROC curve for APPLE score applied on the testing cohort, yielding an** **AUC of 0.557 (95% CI 0.506-0.607).** AUC: area under the receiver operator curve; ROC: receiver operator curve.

**
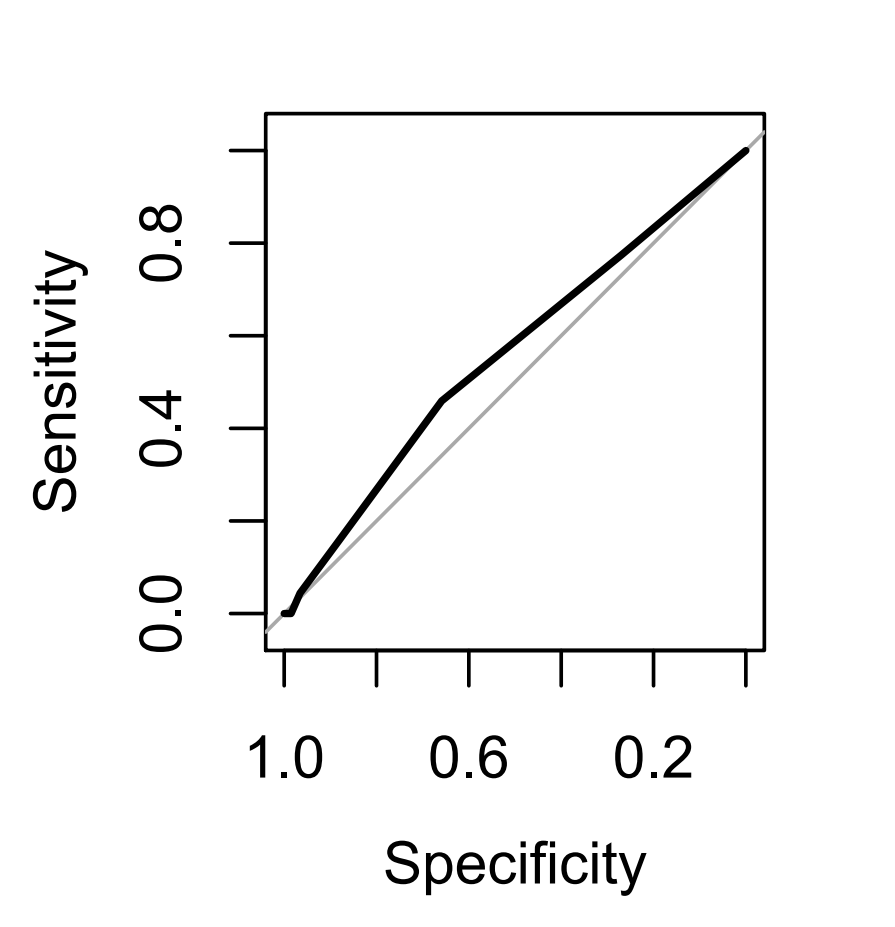
**
